# Researches on the Mechanism of the Human Voice

**Published:** 1833-01-01

**Authors:** 


**Recherches sur le Méchanisme de la Voix Humaine: Ouvrage qui a obtenu un Prix a la Société des Sciences Physiques et Chimiques de Paris. Par *F. Bennati*, M.D. &c. Paris, 1832.**

Physiologists have studied, with peculiar attention, the formation of the voice in the larynx, and its subsequent modification by the tongue, lips, teeth, &c. which constitutes speech; but they have not investigated with similar care its various modulations in singing, when its full powers are called into action. In the search for instruments, the work of human hands, to which this most complicated piece of animal mechanism might be compared, the organ in which undoubtedly sound is produced has been too exclusively examined, and those parts have not been taken into sufficient account, which, from their intimate connexion with the larynx, must exert some considerable influence on the air which passes through them, after having been thrown into vibrations by the chordæ vocales. One physiologist has compared the organ of voice to a wind, another to a stringed instrument, and others to musical instruments combining both these powers; flutes, hautboys, violins, reeds, French horns, Æolian harps, and Æolins have, in succession, served as illustrations to explain the structure and action of an organ composed of cartilages, ligaments, muscles, and glands, lined by mucous membrane, which is constantly kept in a moist state by its peculiar secretion, and preserved in action only by a proper supply of nervous influence and by life. The pithy remark of Dr. Hunter, after enumerating the various cooking machines to which the stomach was compared, that "the stomach, gentlemen, is a stomach," will apply, perhaps, with equal truth to the larynx; nor can we see the force of the objection, that this mode of disposing of a subject, if followed up, would crush all investigation whatsoever; it merely laughs at absurd comparisons, and at attempts to explain the action and structure of vital machines, by reference to those of human workmanship, which have no similarity of structure or mode of action. Dr. Bennati considers that, in order to study with precision the mechanism of the voice in singing, the observer must be both a physiologist and a musician; that he must have paid particular attention to singing; must possess an organ of voice which enables him to make experiments constantly on himself, and that, from his peculiar acquaintances and from travelling, he must have been able to examine the organs of the most celebrated vocalists of the day. Fortunately, Dr. B. unites in himself these qualifications, and, according to

Cuvier's Report, " a reçu de la Nature l'une des voix les plus étendues que l'on connaisse." In a note to a translation of a paper by Dr. Rusch, M. Bennati mentions that he was the pupil of the celebrated Pachierotti, so that he has not neglected the study of a voice which compasses three octaves. He has been engaged during 12 years in these researches, during which time he has constantly examined, not only his own organ of voice, but those of the most celebrated singers. The results he has embodied in a memoir read before the Royal Academy of Sciences, which was referred to Cuvier, Savart, and Prony. The report is drawn up by Cuvier; and, as it contains a lucid explanation of M. Bennati's views, as well as a summary of the information possessed by us on this point previously to this publication, we cannot do better than commence by making some extracts from it.

The principal object of this memoir is to explain the function of an organ in the modulation of the voice, to which physiologists have paid but little attention, that is, the velum palati, or rather that part of the throat bounded superiorly by the velum, inferiorly by the base of the tongue, and laterally by the arches of the palate. Fabricius, of Aquapendente, a man of genius, whose works are at the present day too much neglected, mentions the contraction of the arch of the palate during the formation of acute sounds, and its relaxation under opposite circumstances. The explanation which he gives of the action of the muscles in producing this is erroneous, but the phenomenon is correct. Dodart does not even mention the fauces. Ferrein, after having placed the organ of the voice in the inferior ligaments of the glottis, makes a restriction, and allows that the chordæ vocales are not the organs of every kind of voice, but that there is a voice of the throat, and a treble of the same nature, which are produced by a new organ which he has discovered, and which he promises at a future time to explain; but, although he lived 30 years after this, no other paper on this subject appeared. Haller supposes that Ferrein referred to the velum palati, the motion of which, he does not doubt, must influence the modulation of sounds; but Haller does not enter into particulars, nor have subsequent physiologists been more precise. Such was the information on the action of the parts of the mouth in the modulation of sounds, when M. Bennati, who joins to his professional knowledge great practice in singing, and a voice of the most extensive compass, undertook his researches. He is satisfied that the tongue itself, by being elevated or depressed, or curved into a channel, exercises a powerful influence on the modulations of the voice, and that, before the larynx can produce any intonation whatever, it is necessary that the os hyoides should be fixed in a certain position. He has ascertained, besides, that the notes improperly said to come from the head (voce di testa), or the treble, are owing almost exclusively to the strongest contraction of this upper part of the vocal tube, and he calls them, in consequence, super-laryngeal notes (" notes sur-laryngiennes"), and their union " second registre," to distinguish them from the notes said to come from the chest (la voce di petto), which he prefers calling laryngeal notes, and the whole of which he calls first " registre."*

* The word " registre" is usually employed to denote the stop of an organ. We shall adopt M. Bennati's own term, which with this explanation, will perhaps be the best understood.

He does not mean to say that the larynx does not act in the one, nor the fauces in the other, but only that the fauces play the most essential part in the notes of the second "registre." As to a third registre, which is mentioned in some systems of singing, he regards it as imaginary, and owing only to greater or less vibration of the last notes of the first, and of the first of the second. Those singers whose voice is formed of the two registres require more art to pass from one to the other, so as to unite them, and are fatigued more easily than the others. In the soprani-sfogati, who, by means of the second registre, pass the ordinary scale of soprano, the edges of the tongue become elevated, so as to form a semiconical cavity. In perfect soprani, whose voice is almost exclusively modulated by the first registre, the superior surface of the tongue is, on the contrary, rounded by the depression of its edges, and what is not less remarkable, it is one-third more voluminous than in ordinary individuals. It is to this influence of the tongue, that Dr. Bennati refers the greater or less fitness of different languages for music, according as the motions which are required by the more or less frequent return of certain letters agree, or are opposed, to those which are made by the tongue, and the anterior part of the mouth, for the projection of the note. In grave sounds, at the same time that the larynx is lowered, the velum is elevated and carried backwards, and the uvula is shortened, and becomes thicker. The contrary takes place in acute sounds; whilst the larynx is raised, the velum is depressed and carried forwards, the uvula becomes doubled on itself, and in the most acute notes of the second registre, it disappears entirely. Thus, the tenors-contraltini and the soprani-sfogati have these parts much more developed than the counter-tenors, and there are proportionate differences between the singers of other parts. Those who employ frequently the notes of the second registre, experience a feeling of fatigue at the velum, and if inflammation is the consequence it is confined to those parts; whilst those who make use in singing of the notes of the first registre feel fatigue in the thorax, and are more subject to inflammatory affections of the chest.

M. Bennati concludes by this proposition, that the muscles of the larynx are not the only ones which modulate the voice, but also those of the os hyoides, tongue, and soft palate; that, therefore, the organ of the voice is an organ *sui generis*, inimitable by art. This, although not entirely new, is an observation which M. Bennati has strengthened by fresh proofs; and M. Cuvier proposes that the Academy should testify their satisfaction to the author.

Dr. Bennati commences his memoir by stating, that the object of his researches is not the study of the formation of voice, nor the action by which it becomes speech, nor that more complicated one by which the inflection is modulated during declamation, but the modulation it undergoes during singing, which is distinguished not only by the calculated and harmonic succession of intervals, and the infinite variety of intonations, but especially on that property of song, of existing independently of speech, that is to say, of forming a complete discourse by the vowels, more or less modified. This high degree of modulation, which constitutes singing, is that which requires the most decided exertion, and the most numerous means of modification. Hitherto physiologists have, in accounting for grave and acute sounds, taken into account merely the internal muscles of the larynx, with the hyothyroideus and sterno-thyroideus, which raise or depress it; they have omitted the investigation of the muscles of the os hyoides, of the tongue, and of the superior part of the vocal tube. Meckel has observed, when speaking of the action of the hyo-thyroid muscle, that it serves to elevate the larynx when the os hyoides is fixed above, but Dr. Bennati not only agrees with this, but asserts that the os hyoides is fixed during each sound, in order to facilitate the contraction of the muscles of the larynx, and, consequently, to bring out the notes. Indeed, if the muscles of the os hyoides were cut, or only paralyzed, the larynx, abandoned to its own muscles, would produce merely imperfect and monotonous sounds, both weak and cracked. These remarks are not merely hypothetical, but are founded on experiments on different singing animals, as well as on pathological facts, which it is the intention of Dr. B. to publish. The muscles which elevate the os hyoides in the modulation of the voice are the thyro-hyoidei, mylo-hyoidei, genio-hyoidei, and stylo-hyoidei, they act simultaneously with the major part of the muscles of the tongue, principally with the stylo-glossi, which, at the moment of their contraction, are assisted by the digastrici by means of an aponeurotic expansion, which passes from the tendon of these muscles to be inserted in the os hyoides. The genio-glossus, the linguales, and the hyo-glossus, particularly that portion of it which arises from the lesser cornu of the os hyoides, assist in its elevation. The influence of the tongue in modulation is apparent, even by the simple examination of the relations which exist between its muscles and the os hyoides, and between this bone and the larynx. Moreover, if the motions of the tongue are examined with attention during singing, it will be seen, during the acute notes, to contract at its base, and at the same time to become enlarged; and, in the highest exertion of the second "registre" of soprani-sfogati, its edges will become raised, so as to form a semi-conical cavity, the apex of the cone corresponding to the tip of the tongue. In perfect soprani, that is to say, in those gifted with a round, sonorous, and modulated voice, almost exclusively of a single registre, the tongue is elevated, contracted at its base, enlarged, with its surface slightly rounded by the depression of its edges. In general, during grave notes, the action of the tongue is less marked, and preserves almost entirely its position and its ordinary form, with the exception sometimes of a slight undulation. M. Bennati has derived these facts from the examination of the best singers of the day. In Mademoiselle Sontag, who is the most striking example, at the present time, of a voice of the highest pitch, and a facility of modulating the notes of the second registre, this cavity of the tongue is more marked than in any other soprano. A fact no less singular is, that among singers whose voice is very sonorous, and confined almost exclusively to a single registre, the volume and dimension of the tongue are one-third greater than ordinary. Catalani, Lablache, and Santini are examples. The tongue of the last is the longest and largest that Dr. Bennati has ever seen; even when the genio-glossus is at its maximum of contraction, Santini can touch beneath his chin with the point of his tongue; in the acute notes its extremity is folded on itself, having nearly the form of a hook. Dr. B. has observed, in many singers, that the motions of the lower jaw, lips, and tongue, as well as sometimes certain grimaces of the face during singing, correspond, in some measure, to the internal motion of the muscles which constitute the vocal apparatus. From the striking coincidence which is met with, on this head, in individuals whose voices are strong, sonorous, and of great compass, although confined to the first "registre," he concludes that this combination of motions is, most frequently, only one of the consequences of the ordinary mechanism of the voice. In confirmation of this, the following case is related. Having frequently remarked that the Countess of M., a very distinguished amateur singer, when singing a certain note, inclined her mouth to the left side, Dr. Bennati was anxious to discover whether this arose from a bad habit, or from the peculiar mechanism of the organ. He therefore examined the superior part of the vocal tube whilst the lady sang the particular note, and he found that the motion of the mouth, and of the lower jaw, depended on the mechanism of the tongue, which instead of exhibiting the semi-conical cavity in its middle, presented it on its left side, that is, the side to which the tongue was drawn by the action of its muscles. As Dr. B. speaks many languages, he has studied the difference which the same musical phrase will produce when sung in these various tongues. This difference, when each language is equally well pronounced, is enormous, to the ear, if the Italian is compared with the English; it is progressive in difficulty according to the following order; Italian, Portuguese, Spanish, French, German, English. Thus their difficulty is the inverse of their melody and their charm. This is owing to the particular motions of the tongue and velum palati, as well as to the different modifications which the vocal tube undergoes.

But to return to the larynx. As soon as it is elevated, the simultaneous action of the hyo-thyroidei laterales, hyo-arytenoidei obliqui et transversales, thyro-arytenoidei, together with the contraction of the "thyro-aro-et glosso-epiglottiques," shorten the laryngeal cavity and the trachea. The air from the lungs being driven through the larynx in this state produces a sound more or less acute, according to the vocal power of the individual, but, to take a mean, not passing the "*sol*," the extreme limit beyond which it is impossible to pass, by the action of the above-mentioned muscles alone. The depression of the larynx is effected by the action of the sterno-thyroidei, sterno-hyoidei, and omo-hyoidei, and the larynx at the moment of the modulation is enlarged at the same time by the contraction of the crico-thyroidei, and of the crico-arytenoidei posteriores. In this instance, in expelling the air with more or less force, a grave sound is produced, which, by the assistance of the muscles alone, will compass "*do*," as a mean. Notwithstanding, it is known that the tenors-contraltini (or those whose high notes pass by means of the second registre the ordinary scale of soprano) and the soprani-sfogati in high notes can reach four notes more, or eight demi-tones; and baritenors and base singers in the low notes, can compass four notes more, or eight demi-tones. Since then, whether the larynx is elevated and contracted, or depressed and enlarged, it is insufficient to compass so extensive a series of modulated sounds, it is natural to conclude that it does not constitute the whole vocal apparatus. This, indeed, some physiologists have suspected. M. Savart explains very ingeniously the formation of the human voice; he shews that its production is analogous to that of the sound in the tube of a flute, and that the small column of air contained in the larynx, and in the mouth, is calculated, as well by the nature of the elastic walls which contain it, as by the manner in which it is thrown into vibrations, to produce sounds of a particular character, and at the same time much lower than its dimensions would seem to permit. He has proved further, by numerous experiments on the voice of birds, that a mass of air contained in a tube whose sides are elastic or muscular can produce much lower sounds than those it could effect if the sides were solid. Following up the idea that the sound is at first produced in the ventricles, he endeavours to prove that the air which is contained in the vocal tube, ought always to resound in unison with the sound formed originally in the ventricles, and consequently that it should strengthen that sound in a decided manner. But as M. Savart only proposed to explain the mechanism of the human voice, he has not carried his researches further. Dr. Bennati has therefore endeavoured to examine the modifications, which under the influence of the will, the vocal muscles produce on the form and tension of the vocal tube, so as to strengthen in the most advantageous manner the sounds produced in the ventricles. To effect this object, it was simply necessary to observe the motions which were produced during singing, an effort requiring the strongest powers of the organ. He paid attention chiefly to the upper parts of the vocal tube. In grave sounds, he observed, that the soft palate was elevated and carried backwards, so as to take an arched form, by the action of the circumflexus palati and levator palati of the constrictor isthmi faucium, palato pharyngeus, mylo and genio-hyoideus, together with the styo-glosso-pharyngeus, which act simultaneously with the depression of the larynx; the uvula retains nearly its ordinary position, except that by the contraction of its azygos muscle it becomes thicker and shorter. Thus both the internal movement of the isthmus faucium, and that of the larynx combine during the production of grave sounds to have a freer course to the air, so as to give more volume and extent to the gravity of the sounds. Again, because grave sounds require more air than acute ones, and because expiration is more difficult to be regulated when the vocal tube has acquired its highest degree of shortening and of enlargement, the disposition of the parts is the least favourable for hindering the exit of the air. The contrary phenomena are witnessed during the singing of high notes. Then the velum palati, after being elevated, is depressed and carried forwards by the more vigorous contraction of the muscles previously enumerated in the modulation of grave notes; as in the latter instance these muscles acted from before, backwards, simultaneously with the depression of the larynx, so in the former they act from behind forwards at the same time as the larynx is elevated. The tonsils appear to swell out and approach each other; the uvula from the stronger contraction of its azygos muscle is folded entirely on itself, and in the most acute notes of the second registre entirely disappears. The aperture of the soft palate loses that arched form into which it was thrown during grave notes, and appears like a triangle, slightly blunted at the apex. Singers with voices of great compass, particularly in acute notes, as Dr. Bennati has observed in the first tenors-contraltini of the age, David and Rubini, and in the most celebrated soprani-sfogati, as Mesdames Mombelli, Fodor, Lalande, Catalani, Sontag, Tosi, and others, have the superior part of the vocal tube infinitely more developed and more moveable than the counter-tenors, such as Lablache and Ambroggi. Dr. Bennati has lately examined the mechanism of the voice during ventriloquism, by means of a peculiar speculum. He finds that a ventriloquist always speaks with his superlaryngeal voice, which is peculiarly modified by a very curious elevation of the base of the tongue towards the roof of the palate, whilst its point serves for the articulation of the words which the ventriloquist is accustomed particularly to employ. According to the nature of the voice is the feeling of fatigue which singers experience. Thus, Mesdames Mombelli, Fodor, Sontag, Tosi, among the soprani-sfogati; David, Rubini, Gentili, and many others among the tenors-contraltini, are never more fatigued than after having sung those parts where the notes of the second registre are most frequently employed. This feeling of fatigue extends to the parts which compose the upper part of the vocal tube, without going beyond them. If this is increased by constant or forced exertion, a debility of the nervous system of these parts will be produced, or an inflammation, which sometimes will extend to the trachea, but which rarely affects the bronchia, pleura, or lungs. On the other hand, Lablache, Galli, Ambroggi, Santini, Nozzari, Crivelli, Mesdames Marianni, Catalani, and many others whose voices are almost exclusively confined to the first registre, although they may be of different kinds, after considerable exertion feel fatigue in the diaphragmatic and thoracic regions. If they continue to sing, this feeling of malaise may terminate in an inflammation attacking either the trachea, bronchial tubes, lungs or pleura. In this class of singers nervous debility of the parts composing the summit of the vocal tube is very rare. When there is only, in either case, excessive fatigue, astringent gargles and external alcoholic and camphorated frictions may hasten the cure which repose alone can effect. But as debility of these parts may be easily mistaken for inflammation from the similarity of the symptoms, great care must be taken in the diagnosis, for in treating a case of debility by either local or general antiphlogistic remedies, the disease will be augmented and may terminate in complete aphonia, of which, many celebrated singers of both sexes, of the present day, have been the victims. Besides the cases of David, and Mademoiselle Tosi, who have almost lost their voices from a treatment altogether contrary to their pathological condition, Dr. Bennati cites the case of Madame Mainvielle Fodor, whose treatment, carried a little farther, has deprived her of her voice altogether for many years. The following curious pathological fact tends to confirm M. Bennati's views. The Compte de Fredrigotti, one of the most distinguished amateur singers of the day (who is since dead) had an enlargement of the tonsils which prevented him, as he thought, from making use of the full powers of his voice. He consulted a French surgeon of eminence, and according to his advice, submitted to the extirpation of two-thirds of each of his tonsils, in order that his bari-tenor voice, to which he owed so much of his celebrity, might be rendered more clear, facile, and of greater compass. The consequence was, that his laryngeal voice, (or first registre) became more clear and round, and was augmented by two notes, whilst four super-laryngeal notes, or those of the second registre were lost; proving, that an imperfection in the superior part of the vocal tube produced an imperfect action. Dr. B. is at present attending a young pupil of the "Conservatoire de Milan" (M. Carcelli), in whom, from angina tonsillaris, the tonsils have acquired so great a size, that he has found it impossible to sing, because his voice, which previously was of the ordinary compass of tenor, had lost its sonorousness and compass, and only reached ré "asigu," whilst he could, with the second registre reach five notes more than before. Wishing to avoid an operation, Dr. Bennati tried astringents alone. At the end of fifteen days a marked change was produced in the strength and sonorousness of his voice, and he had gained two laryngeal notes; those notes of the second registre which he had acquired, from his enlarged tonsils, remained. The following practical application of these observations is not without its value. Dr. Koreff, to whom Dr. Bennati had communicated his ideas on this subject, had to open or cauterize an abscess of the tonsils. After having employed all the usual means of coming at the part, such as, pressing down the tongue with a spoon, which caused vomiting, Dr. Koreff directed his patient to sing the highest note he could reach, and whilst the superior part of the vocal tube was thrust forward, he applied the caustic with immediate relief. Dr. Bennati concludes his paper by giving some directions as to the management of the voice at puberty, and as this subject is connected with a pathological fact of practical importance, we shall make no apology for introducing it.

It is well known that at the age of puberty a remarkable change takes place in the voice; usually in man it loses a whole octave, unless from some local or general morbid cause, the parts which concur in the production of the voice become weakened. This weakness may also be occasioned by singing, which is very dangerous at this critical period, for it may injure the development of the organ, either by partially or wholly paralysing it, or by causing an inflammation which may produce complete aphonia. The following case is given in illustration. During the whole period of his change of voice, Donzelli, according to the advice of his master, ceased to sing, and he subsequently acquired one of the finest voices of the present day; Donizetti, one of his fellow pupils, at the same period, continued to use his voice, and in consequence lost it.

The succeeding directions for the instruction of the voice, although they come more properly under the province of the singing master, are not without their interest to the physiologist, from their furnishing additional proofs of the power we possess of educating the muscles. After having, in the first place, prepared the ear of the young pupils to relish music, which they must study mechanically until the age of seven years, it is necessary, as soon as they have been taught to open their mouths, and give them the most favourable form for the projection of sounds, to make them execute gently, and with a very slow movement, not whole gamuts, as is the usual custom, but only those notes which they can sound without effort. This lesson should not be continued more than a quarter of an hour, or half an hour at most, daily, according to the constitution of the individuals, lest the organs which supply the necessary wind, the lungs and its appendages, should suffer. By these means, the vocal muscles will be so trained as to act under the influence of the will, and, when arrived at their full development, will have more flexibility and strength. This want of suppleness and of elasticity is felt by those who have not commenced the study of singing at an early age. Crivelli, an excellent bari-tenor, who had not devoted himself to singing until his thirty-fourth year, could never, notwithstanding all his efforts, reach a note of the second registre. M. Bennati believes that the want of voice, which is so frequently complained of after the fifteenth or twentieth year, is occasioned by the anti-rational instruction of the organ during childhood; the most promising organic conformation being often spoiled by premature and forced exertions.

We have now fulfilled our task, and laid before our readers a complete analysis of Dr. Bennati's work, so as to enable them to judge for themselves whether he has proved the proposition with which he concludes, "that the muscles of the larynx alone are not the only ones which serve to modulate the sounds of the voice in singing, but also those of the os hyoides, of the tongue, and of the superior part of the vocal tube, without the simultaneous and proportionally-combined action of which, the degree of modulation necessary for singing would not take place."

The long period of time during which these investigations have been pursued, the opportunities which Dr. Bennati has enjoyed of examining the most perfect vocal organs, and his possessing so good a practical, as well as theoretical knowledge of music, are strong reasons to entitle his observations and inferences to the attentive consideration of physiologists. The few cases which he has related, and which bear out so fully some of his deductions, make us regret that they are not more numerous; perhaps the nature of the publication prevented him from stating more, and we shall look forward with much pleasure to a more extended work on this subject, in which he has promised to bring forward, not only his experiments on animals, proving several points which now can be regarded only as statements, but also, what are of much greater value, pathological facts, which are the most important bases of physiological deductions. The book is eked out with "reclamations," and observations upon them, but we must leave these touchy gentlemen to settle among themselves the honour of priority. The reporter to the Academy, who cannot be supposed to lean to either side, gives the credit of the modern investigation of these uses of the soft palate, &c. to M. Bennati; and as Baron Cuvier could not collect, from his inexhaustible stores of scientific learning, above a few passages, shewing that the older writers had ever thought of this part in connexion with the voice, we must allow M. Bennati the due merit of having first pointed out and investigated its great, and, we trust, its true importance.

